# Simulated Microgravity Inhibits Rodent Dermal Fibroblastic Differentiation of Mesenchymal Stem Cells by Suppressing ERK/β-Catenin Signaling Pathway

**DOI:** 10.3390/ijms221910702

**Published:** 2021-10-02

**Authors:** Yansiwei Cheng, Yuhao Zhou, Wenjun Lv, Qing Luo, Guanbin Song

**Affiliations:** Key Laboratory of Biorheological Science and Technology, Ministry of Education, College of Bioengineering, Chongqing University, Chongqing 400030, China; chengyansiwei@cqu.edu.cn (Y.C.); zhouyuhao@cqu.edu.cn (Y.Z.); lvwenjun@cqu.edu.cn (W.L.)

**Keywords:** simulated microgravity, mesenchymal stem cells, fibroblastic differentiation, space medicine, skin-repairing

## Abstract

Studies have shown that bone marrow-derived mesenchymal stem cells (BMSCs) can differentiate into dermal fibroblasts to participate in skin-repairing. However, at present, little is known about how microgravity affects dermal fibroblastic differentiation of BMSCs in space. The aim of this study was to investigate the effect of simulated microgravity (SMG) on the differentiation of BMSCs into dermal fibroblasts and the related molecular mechanism. Here, using a 2D-clinostat device to simulate microgravity, we found that SMG inhibited the differentiation and suppressed the Wnt/β-catenin signaling and phosphorylation of extracellular regulated protein kinases 1/2 (ERK1/2). After upregulating the Wnt/β-catenin signaling with lithium chloride (LiCl) treatment, we found that the effect of the differentiation was restored. Moreover, the Wnt/β-catenin signaling was upregulated when phosphorylation of ERK1/2 was activated with tert-Butylhydroquinone (tBHQ) treatment. Taken together, our findings suggest that SMG inhibits dermal fibroblastic differentiation of BMSCs by suppressing ERK/β-catenin signaling pathway, inferring that ERK/β-catenin signaling pathway may act as a potential intervention target for repairing skin injury under microgravity conditions.

## 1. Introduction

Studies have found that astronauts often have skin problems such as allergic rashes and effusion, a significant reduction in elasticity, obvious thinning of the dermis, increased skin sensitivity, and delayed wound healing [1,2,3]. One study showed that skin injuries are the most common medical events in a space mission, including dry and itchy skin that makes it more likely to scratch and irritate [4]. These suggest that under the conditions of microgravity, the normal physiological characteristics of the skin are affected and becomes more vulnerable. Therefore, the prevention and treatment of skin injury and repairing under microgravity conditions has become the focus of space medicine research.

Dermal fibroblasts are critical in supporting skin-repairing [5]. Bone marrow-derived mesenchymal stem cells (BMSCs) are a kind of adult stem cells with multi-directional differentiation ability, which provides a promising method for wound healing and regeneration of damaged tissues [6]. It has been found that BMSCs could differentiate into dermal fibroblasts to participate in wound repairing after skin injury [7,8]. However, little is known about the effect of microgravity conditions on the differentiation process and the involved molecular mechanism.

Wnt/β-catenin signaling can participate in the occurrence of fibrosis and regulate the differentiation of BMSCs into lung fibroblasts [9,10]. However, the role of the Wnt/β-catenin signaling in the dermal fibroblastic differentiation of BMSCs under microgravity conditions has not been fully studied. In addition, Wnt/β-catenin signaling is usually intertwined with other pathways [11]. It has been demonstrated that the regulation of Wnt/β-catenin signaling depends on the phosphorylation of extracellular regulated protein kinases 1/2 (ERK1/2) [12,13]. Studies showed that the phosphorylation of ERK1/2 was inhibited during osteogenic differentiation of BMSCs under simulated microgravity (SMG) conditions [14]. However, it is not clear whether the phosphorylation of ERK1/2 changes and how it affects the Wnt/β-catenin signaling during the differentiation of BMSCs into dermal fibroblasts under the SMG conditions.

Therefore, this study investigated the changes of BMSCs differentiation into dermal fibroblasts under SMG conditions and further revealed the possible molecular mechanism of the changes. Our data suggest that SMG inhibits the dermal fibroblastic differentiation of BMSCs, possibly by suppressing the ERK/β-catenin signaling pathway.

## 2. Results

### 2.1. Dermal Fibroblastic Differentiation of BMSCs

**Figure 1:**
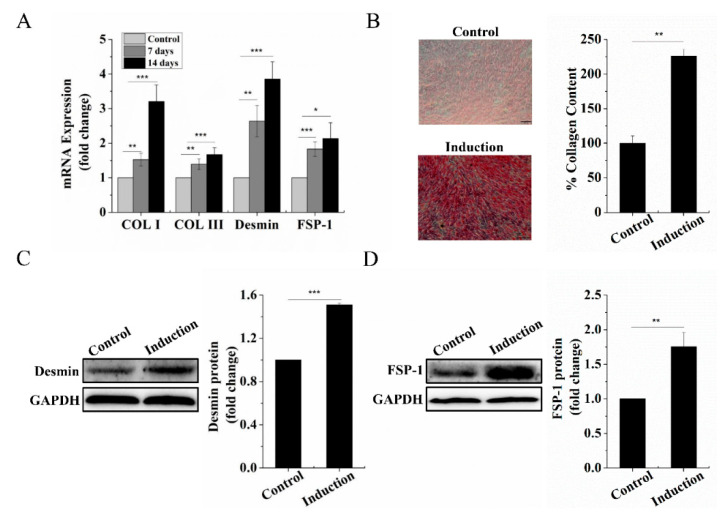
Fibroblastic differentiation of BMSCs upon stimulation with CTGF and ascorbic acid. (**A**) mRNA expression of Col I Col III, Desmin, and FSP-1 after 7 and 14 days of induction by RT-qPCR detection. (**B**) Sirius red staining for Col I and Col III deposition evaluation in BMSCs and quantification data of Sirius red staining after 7 days of induction (scale bar, 100 µm). (**C**) Western blot detection of the Desmin protein level after 7 days of induction. (**D**) Western blot detection of the FSP-1 protein level after 7 days of induction. Data are presented as mean with SD; BMSCs: bone marrow-derived mesenchymal stem cells; CTGF: connective tissue growth factor; Col I: collagen I; Col III: collagen III; FSP-1: fibroblast-specific protein-1; GAPDH: glyceraldehyde-3-phosphate dehydrogenase. *n* = 3, * *p* < 0.05; ** *p* < 0.01; *** *p* < 0.001.

BMSCs can be differentiated into fibroblasts with connective tissue growth factor (CTGF) and ascorbic acid treatment [15,16,17]. Firstly, we evaluated the mRNA expression of collagen I (Col I), collagen III (Col III), Desmin, and fibroblast-specific protein-1 (FSP-1) [17,18], highly specific markers in dermal fibroblasts after treatment of BMSCs with CTGF and ascorbic acid for 7 days and 14 days to confirm differentiation into dermal fibroblastic lineage. We found that the mRNA expression level of Col I, Col III, Desmin, and FSP-1 in induced BMSCs for 7 days and 14 days were significantly increased compared to that of BMSCs without induction, respectively (Figure 1A). This result indicated that 7 days of induction sufficiently prompted BMSCs to differentiate into the dermal fibroblastic lineage. Therefore, in subsequent experiments, we chose 7 days as the induction time. After 7 days of induction, we assessed the deposition intensity of Col I, Col III by Sirius red staining and protein level of Desmin, FSP-1. As expected, the results showed that the deposition intensity of Col I, Col III and quantification data of Sirius red staining (Figure 1B) and protein level of Desmin, FSP-1 (Figure 1C,D) were significantly higher than that of BMSCs without induction, which was consistent with mRNA expression results. Taken together, these results demonstrated that BMSCs can differentiate into dermal fibroblasts induced by CTGF and ascorbic acid.

### 2.2. SMG Inhibited Dermal Fibroblastic Differentiation of BMSCs

**Figure 2:**
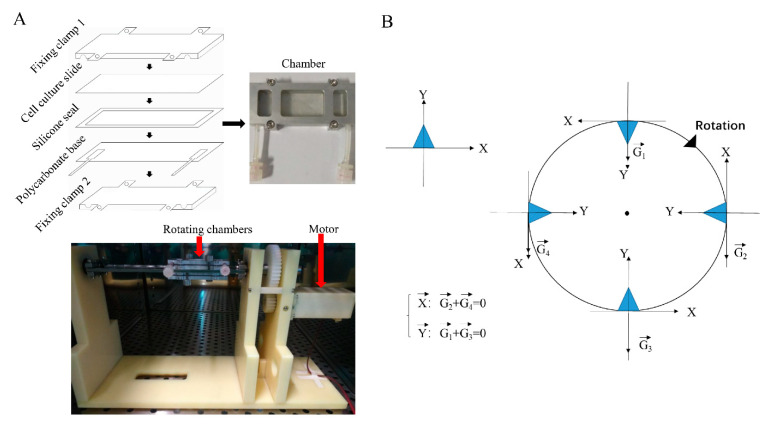
Simulated microgravity rotary flat chamber bioreactor and schematic diagram of BMSCs feeling gravity. (**A**) Construction of a chamber and photograph of the 2D-clinostat in the present study to model simulated microgravity. (**B**) The principle of the clinostat is utilized to simulate microgravity. The rectangular coordinate system based on BMSCs is established. When BMSCs are attached to the cell culture slice, rotation causes that the gravity vector is not recognizable to BMSCs. In other words, the orientation of the cells changes constantly, and the gravity vector changes accordingly. After one rotation, the gravity vector is zero, which means that the average gravity vector is 0 g. BMSCs: bone marrow-derived mesenchymal stem cells.

**Figure 3:**
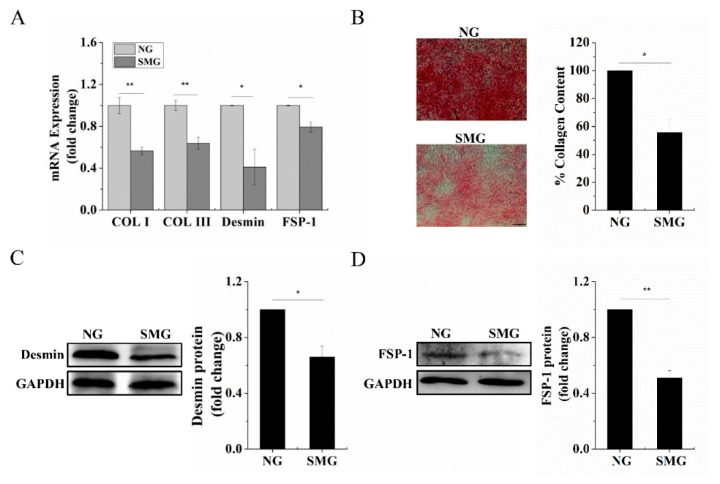
SMG inhibited dermal fibroblastic differentiation of BMSCs after exposure to SMG for 7 days. (**A**) mRNA expression of Col I, Col III, Desmin, and FSP-1 after 7 days of induction under SMG conditions and NG by RT-qPCR detection. (**B**) Sirius red staining for Col I and Col III deposition evaluation in BMSCs and quantification data of Sirius red staining after 7 days of induction under SMG conditions and NG (scale bar, 100 µm). (**C**) Western blot detection of the Desmin protein level after 7 days of induction under SMG conditions and NG. (**D**) Western blot detection of the FSP-1 protein level after 7 days of induction under SMG conditions and NG. BMSCs: bone marrow-derived mesenchymal stem cells; NG: normal gravity; SMG: simulated microgravity; Col I: collagen I; Col III: collagen III; FSP-1: fibroblast-specific protein-1; GAPDH: glyceraldehyde-3-phosphate dehydrogenase. Data are presented as mean with SD; *n* = 3, * *p* < 0.05; ** *p* < 0.01.

The 2D-clinostat device (Figure 2A) was utilized to simulate microgravity in this study. First, we assayed the effect of SMG on dermal fibroblastic differentiation of BMSCs by testing the mRNA expression levels of Col I, Col III, Desmin, and FSP-1. Figure 3A shows that the mRNA expression of the markers above strikingly decreased under SMG conditions compared to that of normal gravity (NG). Similarly, we found that the deposition intensity of Col I, Col III and quantification data of Sirius red staining (Figure 3B) and protein level of Desmin, FSP-1 (Figure 3C,D) were significantly decreased under SMG conditions compared with that of NG. These results indicated that SMG suppressed the differentiation of BMSCs into dermal fibroblasts.

### 2.3. SMG Repressed the Wnt/β-Catenin Signaling and Upregulation of Wnt/β-Catenin Signaling Rescued Dermal Fibroblastic Differentiation of BMSCs

**Figure 4:**
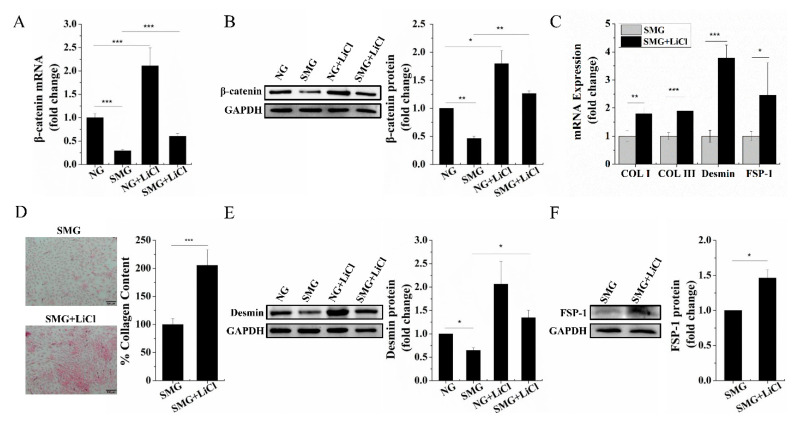
The role of Wnt/β-catenin signaling in the differentiation of BMSCs into dermal fibroblasts under SMG conditions. (**A**) mRNA expression of β-catenin in SMG, SMG + LiCl, NG, NG + LiCl by RT-qPCR detection. (**B**) Western blot detection of β-catenin protein level in SMG, SMG + LiCl, NG, NG + LiCl. (**C**) mRNA expression of Col I, Col III, Desmin, and FSP-1 after LiCl treatment under SMG conditions by RT-qPCR detection. (**D**) Sirius red staining for Col I and Col III deposition evaluation in BMSCs and quantification data of Sirius red staining after LiCl treatment under SMG conditions. (scale bar, 100 µm). (**E**) Western blot detection of the Desmin protein level in SMG, SMG + LiCl, NG, NG + LiCl. (**F**) Western blot detection of the FSP-1 protein level after LiCl treatment under SMG conditions. BMSCs: bone marrow-derived mesenchymal stem cells; NG: normal gravity; SMG: simulated microgravity; Col I: collagen I; Col III: collagen III; FSP-1: fibroblast-specific protein-1; GAPDH: glyceraldehyde-3-phosphate dehydrogenase; LiCl: lithium chloride. Data are presented as mean with SD; *n* = 3, * *p* < 0.05; ** *p* < 0.01; *** *p* < 0.001.

Studies have pointed that Wnt/β-catenin signaling may play a critical role in fibroblastic differentiation [10]. As the central effector in the Wnt/β-catenin signaling, we explored the effect of SMG on the expression of β-catenin during dermal fibroblastic differentiation of BMSCs. We found a marked downregulation of β-catenin expression both at the mRNA (Figure 4A) and protein level (Figure 4B) under SMG conditions compared to that of NG, which revealed that the Wnt/β-catenin signaling was repressed by SMG. Lithium chloride (LiCl), as an activator of the Wnt/β-catenin pathway, can inhibit the activity of glycogen synthase kinase-3 (GSK3), which stabilizes β-catenin [19]. To further explore the relationship between the Wnt/β-catenin signaling and SMG inhibited BMSCs differentiate into dermal fibroblasts, we treated BMSCs with 5 mM LiCl to upregulate the Wnt/β-catenin signaling [20]. As shown in Figure 4A,B, LiCl treatment restored the expression of β-catenin both at the mRNA and protein levels. Then, we investigated the change of the SMG-inhibited dermal fibroblastic differentiation of BMSCs after upregulation of the Wnt/β-catenin signaling. We found that the mRNA expression levels of Col I, Col III, Desmin, and FSP-1 were significantly increased with 5 mM LiCl treatment under SMG conditions (Figure 4C). Additionally, we also found that the deposition intensity of Col I, Col III, and quantification data of Sirius red staining (Figure 4D) and protein level of Desmin, FSP-1 (Figure 4E,F) were remarkably increased compared to that of BMSCs without LiCl treatment under SMG conditions. These findings suggest that upregulation of the Wnt/β-catenin signaling could rescue the SMG-inhibited dermal fibroblastic differentiation of BMSCs, which infer the Wnt/β-catenin signaling is involved in the dermal fibroblastic differentiation of BMSCs under SMG conditions.

### 2.4. SMG Inhibited Phosphorylation of ERK1/2 then Enhancing Phosphorylation of ERK1/2 Improved the Expression of β-Catenin

**Figure 5:**
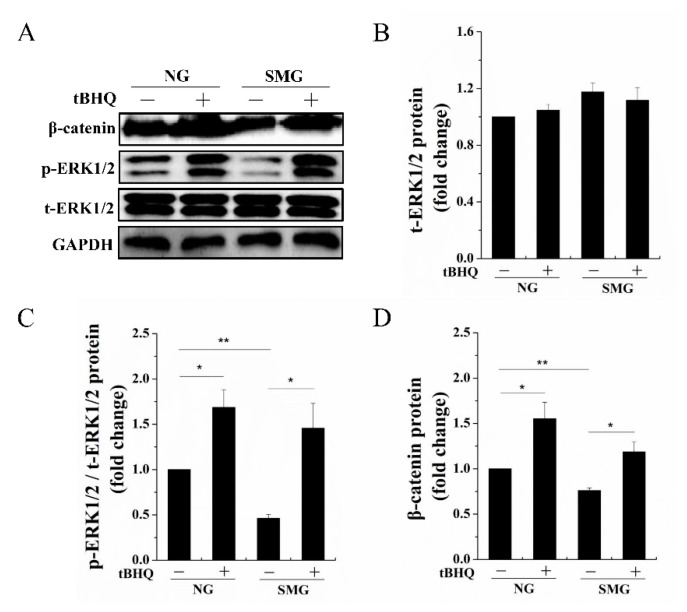
The role of phosphorylation of ERK1/2 in the differentiation of BMSCs into dermal fibroblasts under SMG conditions. (**A**) Western blot detection of the t-ERK1/2, the p-ERK1/2, and the β-catenin protein level in NG, NG + tBHQ, SMG, SMG + tBHQ. (**B**–**D**) Quantification data of Western blot detection of the t-ERK1/2, the p-ERK1/2, and the β-catenin protein level in NG, NG + tBHQ, SMG, SMG + tBHQ. SMG: simulated microgravity; NG: normal gravity; BMSCs: bone marrow-derived mesenchymal stem cells; tBHQ: tert-Butylhydroquinone; t-ERK1/2: total extracellular regulated protein kinases 1/2; p-ERK1/2: phosphorylation of extracellular regulated protein kinases 1/2; GAPDH: glyceraldehyde-3-phosphate dehydrogenase. Data are presented as mean with SD; *n* = 3, * *p* < 0.05; ** *p* < 0.01.

Studies have found that the phosphorylation of ERK1/2 is an upstream signal molecule of the Wnt/β-catenin signaling [12]. Additionally, it was reported that the phosphorylation of ERK1/2 has alterations under SMG conditions in BMSCs [14]. These results lead us to explore a possible signaling pathway on SMG that affects the dermal fibroblastic differentiation of BMSCs. We found that the phosphorylation of ERK1/2 (Figure 5A–C) was evidently reduced under SMG conditions compared to that of NG, which demonstrated that the phosphorylation of ERK1/2 was involved in SMG-inhibited dermal fibroblastic differentiation of BMSCs. Tert-Butylhydroquinone (tBHQ) is an ERK1/2 activator. A previous study showed that the formation of oxidative metabolites derived from tBHQ was responsible for the ERK1/2 activation [21]. To further determine the relationship between the phosphorylation of ERK1/2 and the Wnt/β-catenin signaling during dermal fibroblastic differentiation of BMSCs under SMG conditions, we treated BMSCs with 10 µM tBHQ [22]. As shown in Figure 5, the phosphorylation of ERK1/2 remarkably enhanced, and we also observed that the protein level of β-catenin (Figure 5D) was obviously increased. These finds indicated that SMG suppressed the phosphorylation of ERK1/2 and thus decreased the Wnt/β-catenin signaling during dermal fibroblastic differentiation of BMSCs.

## 3. Discussion

In this study, we have established a differentiation model of BMSCs into dermal fibroblasts and demonstrated that SMG inhibited the dermal fibroblastic differentiation of BMSCs by suppressing ERK/β-catenin signaling pathway. To our knowledge, this is the first study to demonstrate the above findings. Our findings provide a potential target for directing the dermal fibroblastic differentiation of BMSCs to promote skin-repairing for clinical application of skin-repairing in space.

Dermal fibroblasts are the main structural components of the skin dermis, which can synthesize and secrete extracellular matrix such as collagen fibers [23]. They are the most important tissue repairing cells and play an important role in maintaining the strength and integrity of skin [24]. Moreover, BMSCs can migrate to the wound site after skin injury and differentiate into skin fibroblasts and other skin cells to participate in wound repairing [8,25,26]. In light of this, we established the differentiation of BMSCs into dermal fibroblasts with CTGF treatment in vitro. The high expression of Desmin, FSP-1, and fibroblasts ECM components such as Col I and Col III, are known as the dermal fibroblast markers [8,18,27,28]. Thus, Col I, Col III, Desmin, and FSP-1 were selected as indicators to test the differentiation effect after the induction for 7 days. Our results indicated that the expression of these markers was significantly increased after induction, which was consistent with other studies [15,16,17,29].

In recent years, more and more attention has been paid to mechanical stimulation, such as microgravity, in regulating stem cell fate [30]. It has been reported that microgravity can accelerate the differentiation of BMSCs [31,32], such as the differentiation into neurocytes or adipocytes but also can inhibit the differentiation of BMSCs [33], such as the differentiation into osteoblasts. For our experiment, we determined to explore whether microgravity affects the differentiation of BMSCs into dermal fibroblasts. In the present study, a 2D-clinostat was utilized to simulate microgravity on the ground [34]. Our results suggest that SMG suppressed the differentiation of BMSCs into dermal fibroblasts, which is meaningful for providing a possible reference under microgravity conditions in space.

The canonical Wnt/β-catenin signaling pathway regulates cell growth, differentiation, and apoptosis [35]. Sun and colleagues showed that transplanted MSCs differentiated into fibroblasts in the process of inducing pulmonary fibrosis. Meanwhile, the expression of β-catenin was significantly increased. Furthermore, the inhibition of the Wnt/β-catenin signaling pathway decreased the expression of β-catenin and fibroblastic markers in MSCs during the process of inducing fibrosis. Their studies indicated that the canonical Wnt/β-catenin signaling is a key regulator of fibrogenesis and plays a key role in regulating the differentiation of MSCs into fibroblasts [10,36]. Therefore, we assessed whether SMG affects the Wnt/β-catenin signaling during the differentiation of BMSCs into dermal fibroblasts. There exists crosstalk between β-catenin signaling and other pathways [11]. The phosphorylation of ERK1/2 can associate with and primes GSK-3β for its inactivation resulting in upregulation of β-catenin [12]. Furthermore, the phosphorylation of ERK1/2 was inhibited in the differentiation of BMSCs into osteoblasts under SMG conditions [14]. The ERK/β-catenin pathway was found to mediate physiological functions in different cells. Yang and colleagues reported that ERK/β-catenin signaling pathway participated in a positive feedback loop that enhances invasion/migration ability in lung cancer cell lines [37]. Qian and colleagues found the ERK/β-catenin pathway was involved in the immunosuppressive function of splenic stroma-educated regulatory dendritic cells [38]. In this study, our findings suggested that ERK/β-catenin signaling pathway was involved in BMSC differentiate into dermal fibroblasts under SMG conditions.

**Figure 6:**
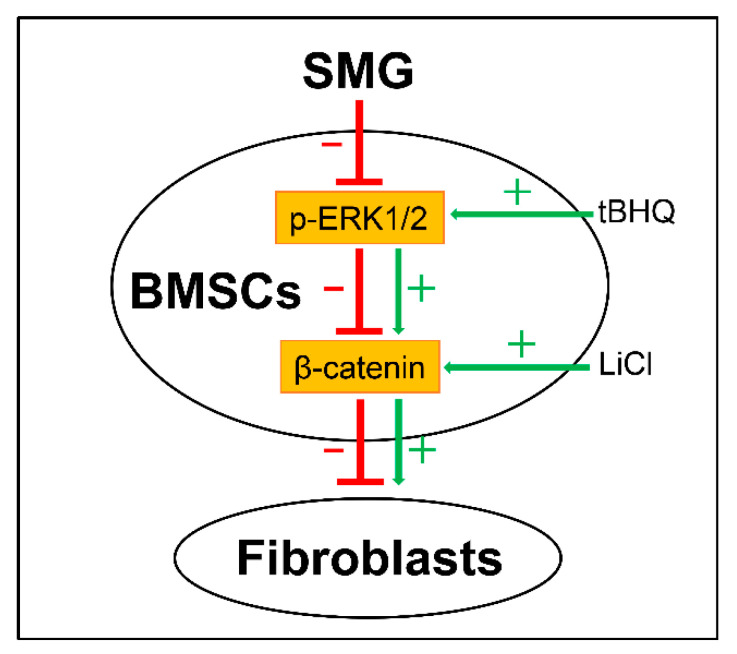
Proposed a model by which SMG inhibits ERK/β-catenin signaling pathway and thus suppresses dermal fibroblastic differentiation of BMSCs. After exposure to SMG, phosphorylation of ERK1/2 was decreased and the Wnt/β-catenin signaling was downregulated, followed by the inhibition of dermal fibroblastic differentiation. After LiCl treatment, the Wnt/β-catenin signaling was upregulated and thus dermal fibroblastic differentiation of BMSCs was recovered under SMG conditions. After tBHQ treatment, phosphorylation of ERK1/2 was enhanced, and thus the Wnt/β-catenin signaling was improved under SMG conditions. Collectively, SMG could suppress ERK/β-catenin to inhibit dermal fibroblastic differentiation of BMSCs. SMG: simulated microgravity; BMSCs: bone marrow-derived mesenchymal stem cells; p-ERK1/2: phosphorylation of extracellular regulated protein kinases 1/2; tBHQ: tert-Butylhydroquinone; LiCl: lithium chloride. “+”/color green lines indicate stimulation; “–”/color red lines indicate inhibition.

Taken together, SMG inhibits the Wnt/β-catenin signaling to impede dermal fibroblastic differentiation of BMSCs, which is possibly due to the decreased phosphorylation of ERK1/2. The upregulation of the Wnt/β-catenin signaling with LiCl treatment reversed the inhibition of SMG on dermal fibroblastic differentiation of BMSCs. Activating the phosphorylation of ERK1/2 by tBHQ improved the Wnt/β-catenin signaling. Our results suggest that both the Wnt/β-catenin signaling and the phosphorylation of ERK1/2 are potential targets for the SMG-inhibited dermal fibroblastic differentiation of BMSCs. An overview schematic diagram (Figure 6) exhibits the proposed model of the inhibition of the dermal fibroblastic differentiation of BMSCs induced by SMG, which shows that ERK/β-catenin signaling pathway mediated the differentiation under SMG conditions. Our findings may provide a basic understanding for spaceflight-induced pathophysiological alterations and help to develop a potential therapeutic target for repairing injured skin for space medicine.

## 4. Materials and Methods

### 4.1. Cell Isolation and Culture

All animal experimental procedures were performed conforming to ethical standards, and national and international standards, and also were approved by Chongqing Science and Technology Commission, Chongqing, China. All Sprague–Dawley (SD) rats, regardless of their gender, used in this study weighing approximately 100 g were provided by Chongqing Medical University. The BMSCs were isolated from the SD rats by the method of whole bone marrow adhesion, as described previously [39]. Briefly, the hind legs of SD rats were completely isolated in a sterile environment. After the muscle tissue was removed, the femurs and tibias of SD rats were separated. Then, the bone marrow cavity was gently blown with a syringe. The BMSCs were cultured in low-glucose Dulbecco’s modified Eagle’s medium (DMEM; Gbico, MA, USA) supplemented with 10% fetal bovine serum (Hyclone, Logan, UT, USA), 2 mM glutamine, penicillin (100 U/mL) and streptomycin (100 µg/mL) in a humidified incubator with 5% CO_2_ at 37 °C. All of the cells used in this study were between passage 2–4.

### 4.2. Dermal Fibroblastic Differentiation of BMSCs

For dermal fibroblastic differentiation, when the cells reached 80%-90% confluence, the culture medium was changed to the differentiation medium supplemented with 100 ng/mL recombinant human CTGF (Peprotech, Rocky Hill, NJ, USA) and 50 µg/mL ascorbic acid (Sigma, Minato City, MI, USA). The differentiation medium was changed every 2 days for 7 and 14 days.

### 4.3. Microgravity Simulation

In this study, the 2D-clinostat device constructed by the National Microgravity Laboratory, Institute of Mechanics, Chinese Academy of Sciences, China, was utilized to simulate microgravity. The clinostat is an effective ground tool for simulating microgravity, the principle of clinostat to model simulated microgravity is to continuously rotate and change the orientation of the cell to gravity to prevent the cell from feeling gravity. As shown in Figure 2B, suppose cells adhered to the slide, when the sample rotates for a full 360°, the average gravitational vector cells sense reduces to approximately 0 g [40]. Cells attached to the cell culture slide (25 × 75 mm), and the distance from the center of the cell culture slide to the rotation axis was 10 mm. The culture slide was located in a closed culture chamber and rotates around the horizontal axis. With rotating in a stable speed, cells in the clinostat would be mechanical equilibrium of gravity and centrifuge forces [41]. In this experiment, when the clinostat rotates at 10 rpm, the apparent gravity on cells can be reduced to about 10^−3^ g, and the centrifugal force generated by the 2D-clinostat was approximately 10^−3^ g too. BMSCs were injected into each chamber at a total count of 3 × 10^5^ cells and all air bubbles were carefully removed to prevent fluid shear stress. After cells were attached to the slide for 24 h and reached 80–90% confluence, the chambers were fixed to the clinostat, and the effect of simulated microgravity was achieved by rotation around the horizontal axis at 10 rpm for 7 days in an incubator with 5% CO_2_ at 37 °C. As normal gravity control groups, static cells were cultured under the same conditions but were not rotated.

### 4.4. Quantitative Real-Time PCR

Total cellular RNA was extracted using an RNA extraction kit (Bioteke, Wuxi, China) according to the manufacturer’s instructions. The total extracted RNA concentration was determined using a Nanodrop 2000 spectrophotometer (Thermo Fisher Scientific, Waltham, MA, USA). Next, 1 µg of the extracted RNA was reversely transcribed to cDNA by using the PrimeScript™ RT reagent kit with gDNA eraser (TaKaRa, Tokyo, Japan). Then, quantitative Real-time PCR was performed using a CFX96™ real-time PCR detection system (Bio-Rad CFX Manager system, Hercules, CA, USA) with 2x SYBR Green qPCR Master Mix (Bimake, San Francisco, CA, USA) according to the manufacturer’s instructions. The PCR reaction conditions used were 5 min at 95 °C, followed by 40 cycles at 95 °C for 15 s and 30 s at 60 °C. The primer sequences are listed in Table 1. β-actin was used as an internal control.

### 4.5. Western Blot

The total protein of the BMSCs was extracted on ice using a RIPA lysis buffer (Beyotime, Shanghai, China) supplemented with a 1% protease inhibitor cocktail (Bimake, San Francisco, CA, USA). Protein concentrations of the lysates were measured with a BCA protein assay kit (Beyotime, Shanghai, China). The lysates mixed with a 5x sample loading buffer were boiled for 10 min at 100 °C for denaturation. Then, 20 µg of each protein sample was separated with 10% SDS-PAGE gel and was electroblotted onto PVDF membranes (Millipore, Billerica, MA, USA). The membranes were blocked for 1 h at room temperature with 5% non-fat milk in Tris-buffered saline (TBS) with 0.05% Tween 20 (TBST). Then the membranes were incubated with primary antibodies (Desmin, Abcam, Cambridge, ab32362, UK), (FSP-1, Abcam, ab197896, UK), (β-catenin, Abcam, ab32572, UK), (t-ERK1/2, Abcam, ab184699, Cambridge, UK), (p-ERK1/2, Cell Signaling Technology, 4370T, Danvers, MA, USA), and (GAPDH, Bioss, bsm-33033M, China) diluted in primary antibody diluents (Beyotime, Shanghai, China) overnight at 4 °C. Next, the membrane was washed 3 times with TBST for 10 min each time and incubated with an appropriate secondary antibody (ZENBIO, Chengdu, China) with 5% non-fat milk for 1 h at room temperature and then washed 3 times with TBST for 10 min each time, the protein expression was visualized using an ECL blotting analysis system (Bio-OI, Guangzhou, China).

### 4.6. Sirius Red Staining

The BMSCs were washed with PBS, fixed by 4% paraformaldehyde (Biosharp, China) for 30 min. Then the BMSCs were rinsed with distilled water 3 times, followed by dyeing with Sirius red staining solution (Solarbo, Beijing, China) for 1 h. Next, the BMSCs were rinsed with distilled water 3 to 5 times until the color was eluted. Digital images were taken using an inverted phase contrast microscope (Olympus, Tokyo, Japan). After imaging, the expression of Col I and Col III was analyzed and quantified by the software ImageJ.

### 4.7. Statistical Analysis

All data were expressed as the mean ± SD. Independent experiments were repeated at least 3 times for each assay. *p* values were analyzed by one-way analysis of variance (ANOVA) followed by two-tailed Student’s *t*-test. When *p* < 0.05, the data between 2 groups was considered to have statistically significant differences.

## Figures and Tables

**Table 1:** Primers sequence for quantitative real-time PCR.

Genes	Forward Primer Sequence (5′-3′)	Reverse Primer Sequence (5′-3′)
COL I	GGAGAGAGTGCCAACTCCAG	GTGCTTTGGAAAATGGTGCT
COL III	TCCCAGAACATTACATACCACT	GCTATTTCCTTCAGCCTTGA
Desmin	CTCTACGAGGAGGAGATGCG	AGGTCAATTCGAGCCAGAGTG
FSP-1	GCACTTCCTCTCTCTTGGTCTG	GTCTTCACTTCTTCCGGGGC
β-catenin	AACGGCTTTCGGTTGAGCTG	TGGCGATATCCAAGGGCTTC
β-actin	ACGGTCAGGTCATCACTATCG	GGCATAGAGGTCTTTACGGATG

## Data Availability

All data are contained within the article.
